# Supplementary material for: Spatial mapping of hepatic ER and mitochondria architecture reveals zonated remodeling in fasting and obesity
Source: Nat Commun. 2024 May 10;15:3982. doi: 10.1038/s41467-024-48272-7 (PMC11087507; doi:10.1038/s41467-024-48272-7)
Supplement: Supplementary file 1 — Supplementary Information [file 41467_2024_48272_MOESM1_ESM.pdf]

Supplementary Figures:

Supplementary Fig. 1

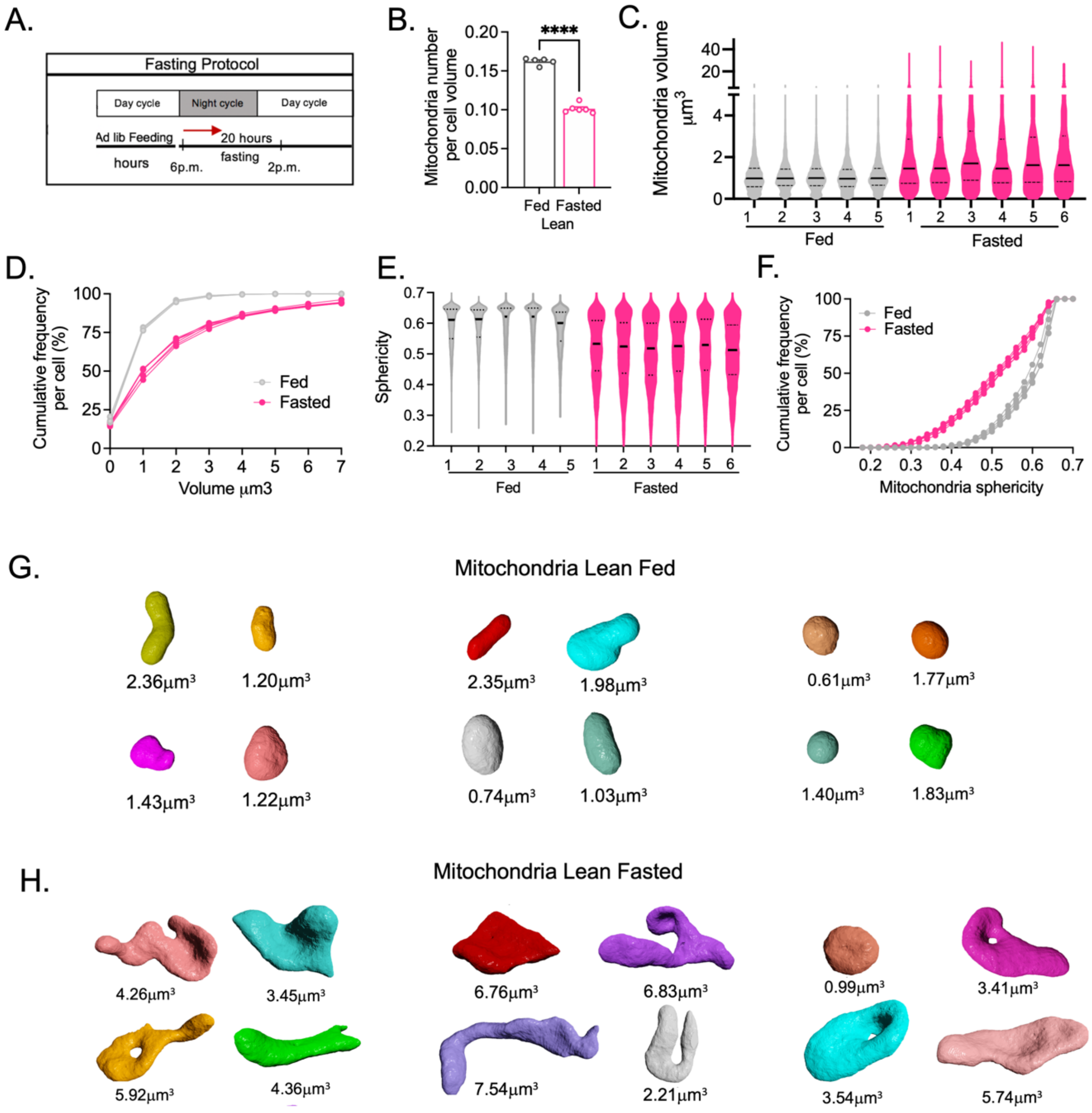

Supplementary Figure 1. Visualization and quantification of mitochondria morphologies from livers from mice in fed and fasted states. (A) Experimental design of the fasting protocol. (B) Number of mitochondria normalized per μm^3 of cell volume (n=5 for fed and n=6 for fasted). (C) Mitochondria volume per cell in livers derived from fed and fasted mice. (D) Cumulative frequency of mitochondria volume per cell in livers derived from fed and fasted mice. (E) Mitochondria sphericity per cell in livers derived from fed and fasted mice. (F) Cumulative frequency of mitochondria sphericity per cell in livers derived from fed and fasted mice. (G, H) Examples of 3D mitochondria morphologies and volumes in fed and fasted states. For the bar graph data is shown as mean ± s.e.m, two-tailed unpaired t-test, ****$p<0.0001$.

Supplementary Fig. 2

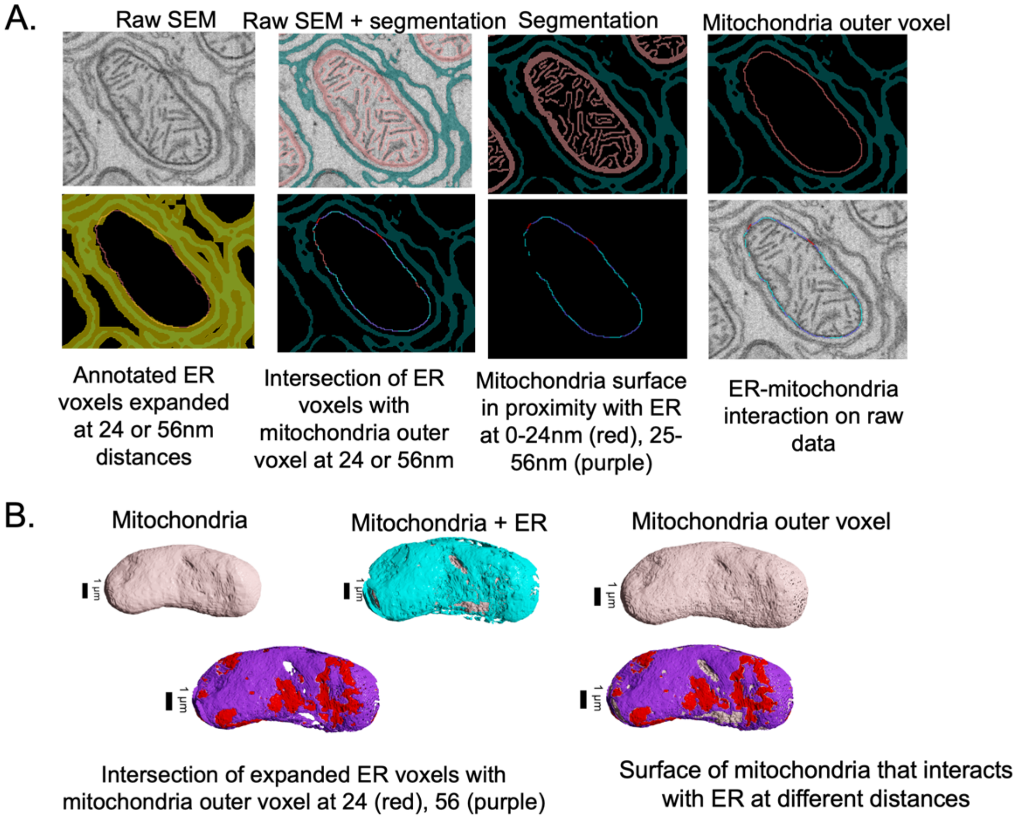

Supplementary Figure 2. Pipeline for quantification of ER-mitochondria interactions in FIB-SEM data sets. (A, B) Workflow for ER-mitochondria interaction quantification in 3D. First, mitochondria outer voxels were generated (single voxel thick). Annotated ER voxels were expanded at different distances (e.g., 24 or 56 nm). The intersection between expanded ER-annotated voxels and the mitochondria outer voxel was computed. To calculate the percentage of mitochondria surface interacting with ER, the interaction surface was divided by the single-voxel-thick mitochondria outer surface. Scale bars: 1µm.

Supplementary Fig. 3

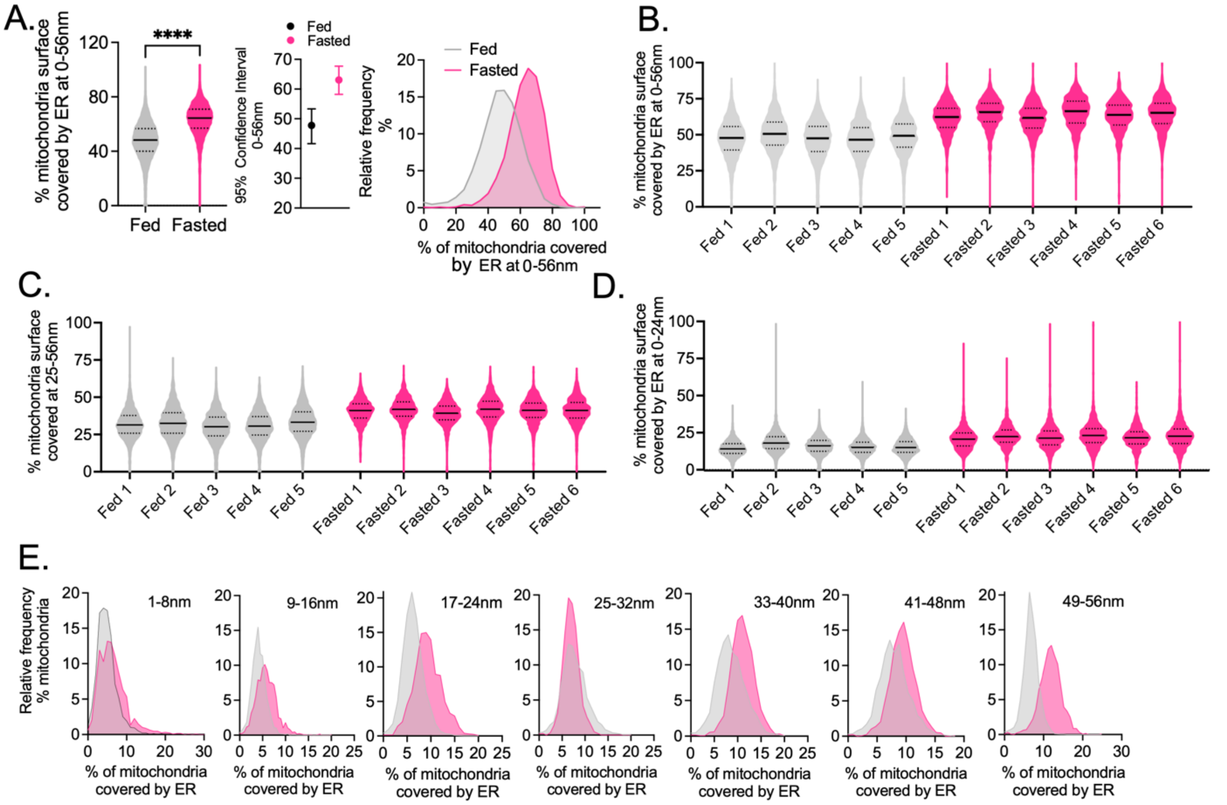

Supplementary Figure 3. Impact of fasting on hepatic ER and mitochondria interaction. (A) Left: Quantification of the mitochondria surface covered by ER at 0-56nm distance in fed (n=14,855 mitochondria) and fasted (n=6,678 mitochondria) conditions. Middle: 95% of confidence interval for data in A. Right: Frequency distribution of percent of mitochondria covered by ER at 0-56nm distance. (B) Quantification of the mitochondria surface covered by ER at 0-56nm distance per cell in fed and fasted livers. (C) Quantification of the mitochondria surface covered by ER at 24-56nm distance per cell in fed and fasted livers. (D) Quantification of the mitochondria surface covered by ER at 24-56nm distance per cell in fed and fasted livers. (E) Pixel-by Pixel (as indicated in the figure) frequency distribution of percent of mitochondria covered by ER. For B, C, D: Fed (n = 2996, 3173, 3109, 2787, 2774), Fasted (n = 894, 1182, 1275, 1246, 757, 1323). For E: Fed (n= 2766) and Fasted (n=1275). In A, two-tailed, unpaired t-test ****p<0.0001, and permutation test ****p<0.0001.

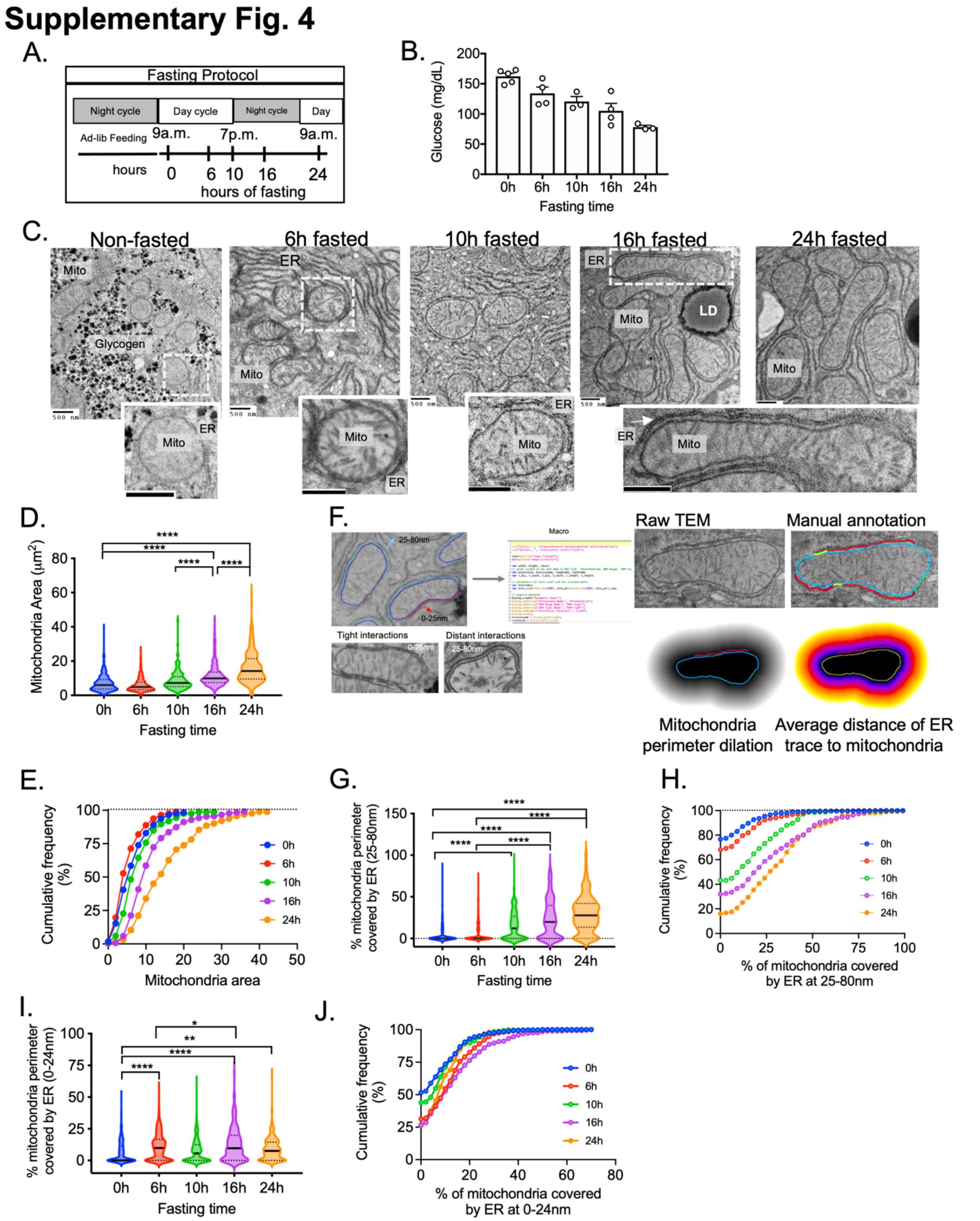

Supplementary Figure 4. Impact of fasting time-course on hepatic subcellular architecture. (A) Fasting time-course experimental protocol. (B) Blood glucose levels of mice at indicated fasting timepoints. (C) Representative TEM images from livers at indicated fasting time points.

Scale bars: 500nm. (D) Quantification of mitochondria area from TEM images at indicated timepoints (n=9 for 0h, n=8 for 6h, n=6 for 10h, n=7 for 16h, n=7 for 24h). (E) Cumulative frequency of mitochondria area from data shown in D. (F) Workflow used for the quantification of ER-mitochondria interactions from 2D-TEM images. (G) Percent of mitochondria surface covered by ER at 25-80nm distance, quantified from TEM images at indicated fasting time points (n=502 for 0h, n=505 for 6h, n=317 for 10h, n=292 for 16h, n=251 for 24h). (H) Cumulative frequency of mitochondria area from data shown in G. (I) Percent of mitochondria surface covered by ER at 0-24 nm distance, quantified from TEM images at indicated fasting time points (n=502 for 0h, n=505 for 6h, n=317 for 10h, n=292 for 16h, n=251 for 24h). (J) Cumulative frequency of mitochondria area from data shown in I. For the bar graph data is shown as mean ± s.e.m.; For D, G and I, one-way Anova and Tukey's multiple comparisons test. *p<0.0408, **p=0.005, ****p<0.0001.

Supplementary Fig. 5

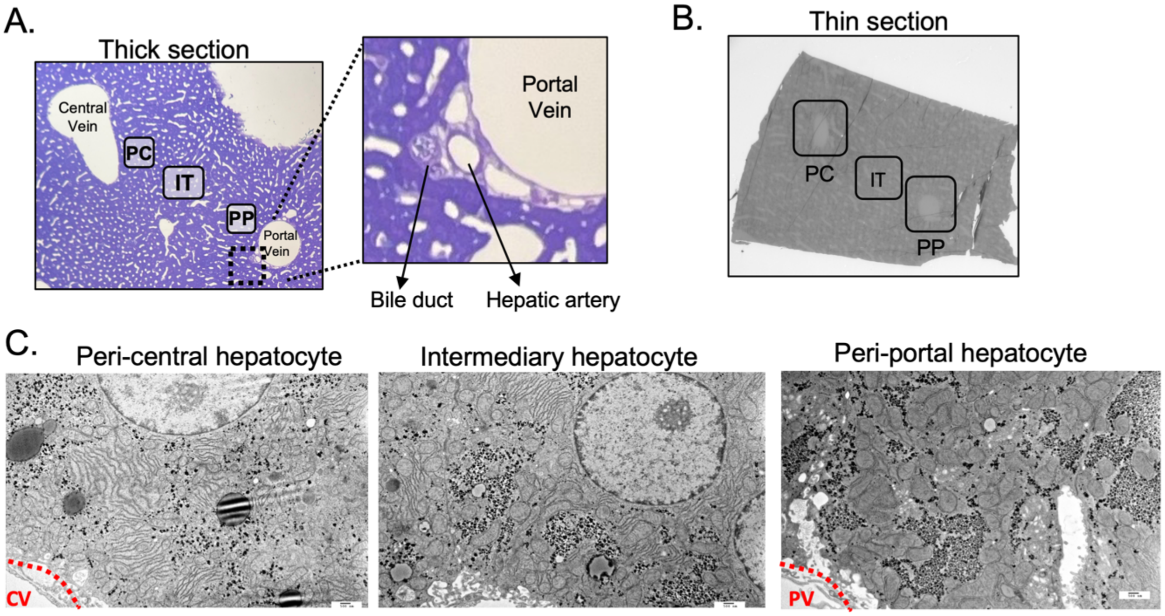

Supplementary Figure 5. Identification of distinct hepatic zones within the liver lobe. (A) Histological sections stained with Hematoxylin and Eosin (H&E) depicting the central vein and the portal vein region surrounded by hepatic artery and bile duct. Pericentral (PC), mid-lobular (IT) and periportal (PP) hepatocytes are indicated. (B) Transmission electron microscopy view of the histological section on the left, used as an anatomical guide to choose the regions of interest. (C) Representative TEM images from indicated hepatic zones. Scale bars: 500nm.

Supplementary Fig. 6

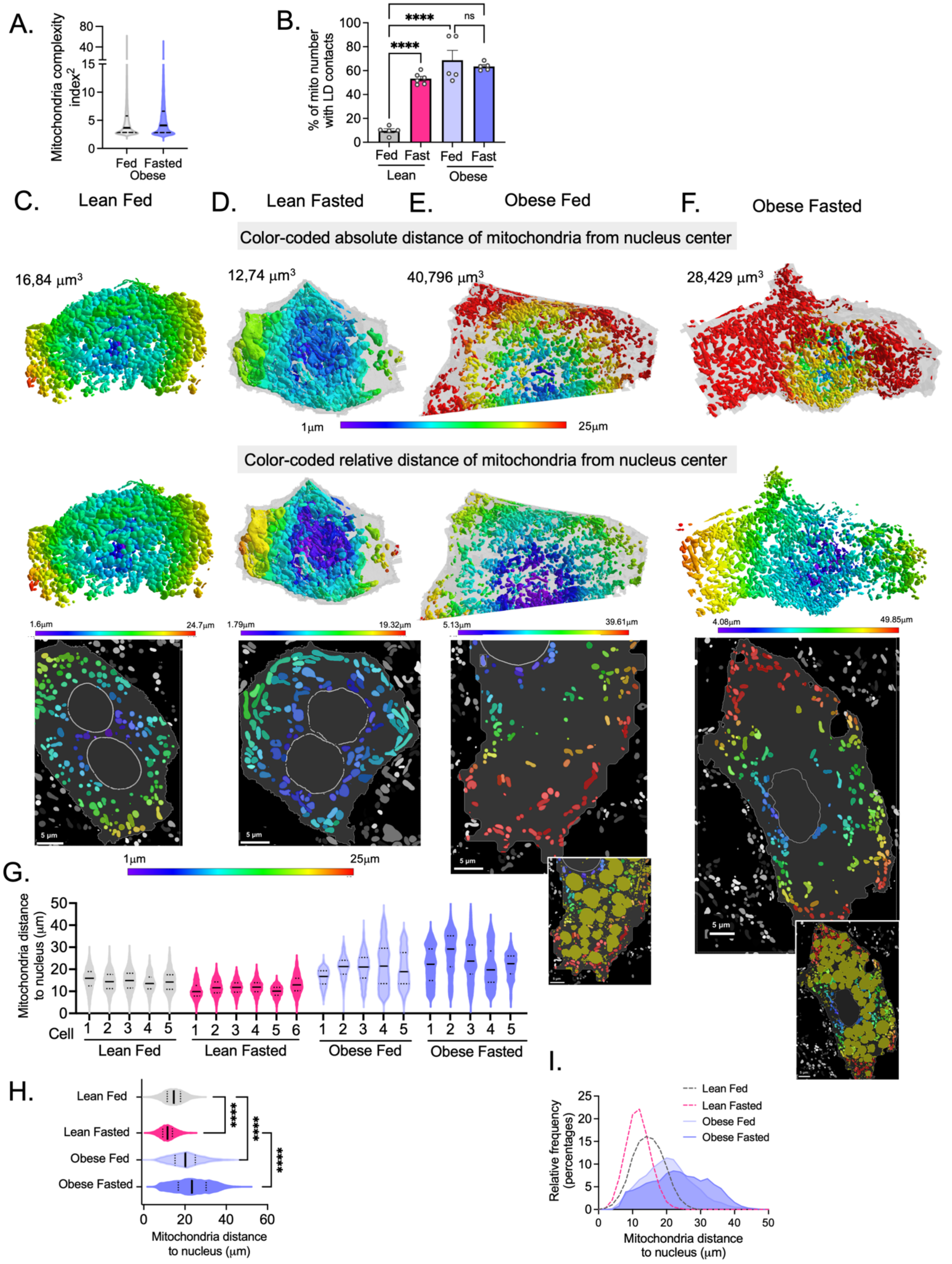

Supplementary Figure 6. 3D subcellular distribution of mitochondria in fasting and obesity. (A) Mitochondria complexity index (MCI^2) of mitochondria from obese livers in fed (n=17721) and fasted (n=12711) state. (B) Quantification of percent of mitochondria interacting with lipid droplets (LD) (n=5 cells for lean fed, obese fed, obese fasted and n=6 cell for lean fasted). (C-F) Subcellular spatial distribution of mitochondria. The distance from the center of 3D geometry of each mitochondrion to the center of the nucleus was color-coded based on its absolute distance (top) and relative distance (middle). The bottom images show mitochondria distribution in a 2D cross-section of the cells. Scale bars: 5µm. (G-H) Quantification of mitochondria distance to the nucleus center in 3D, cell by cell (G) and per average of the indicated group (H). (I) Relative frequency distribution of percent of mitochondria distance to nucleus center in 3D. For G, lean fed (n = 3001, 3175, 3111, 2790, 2778), lean fasted (n = 895, 1185, 1277, 1248, 759, 1325), obese fed (n= 2085, 5056, 3856, 3522, 3202) and obese fasted (n= 3252, 2783, 2609, 2067, 2001) states. For (H, I), lean fed (n= 14855), lean fasted (n=6689), obese fed (n=17721), obese fasted (n=12712) states. For the bar graph data is shown as mean ± s.e.m; For B an H, one-way Anova and Tukey's multiple comparisons test. ****p<0.0001.

Supplementary Fig. 7

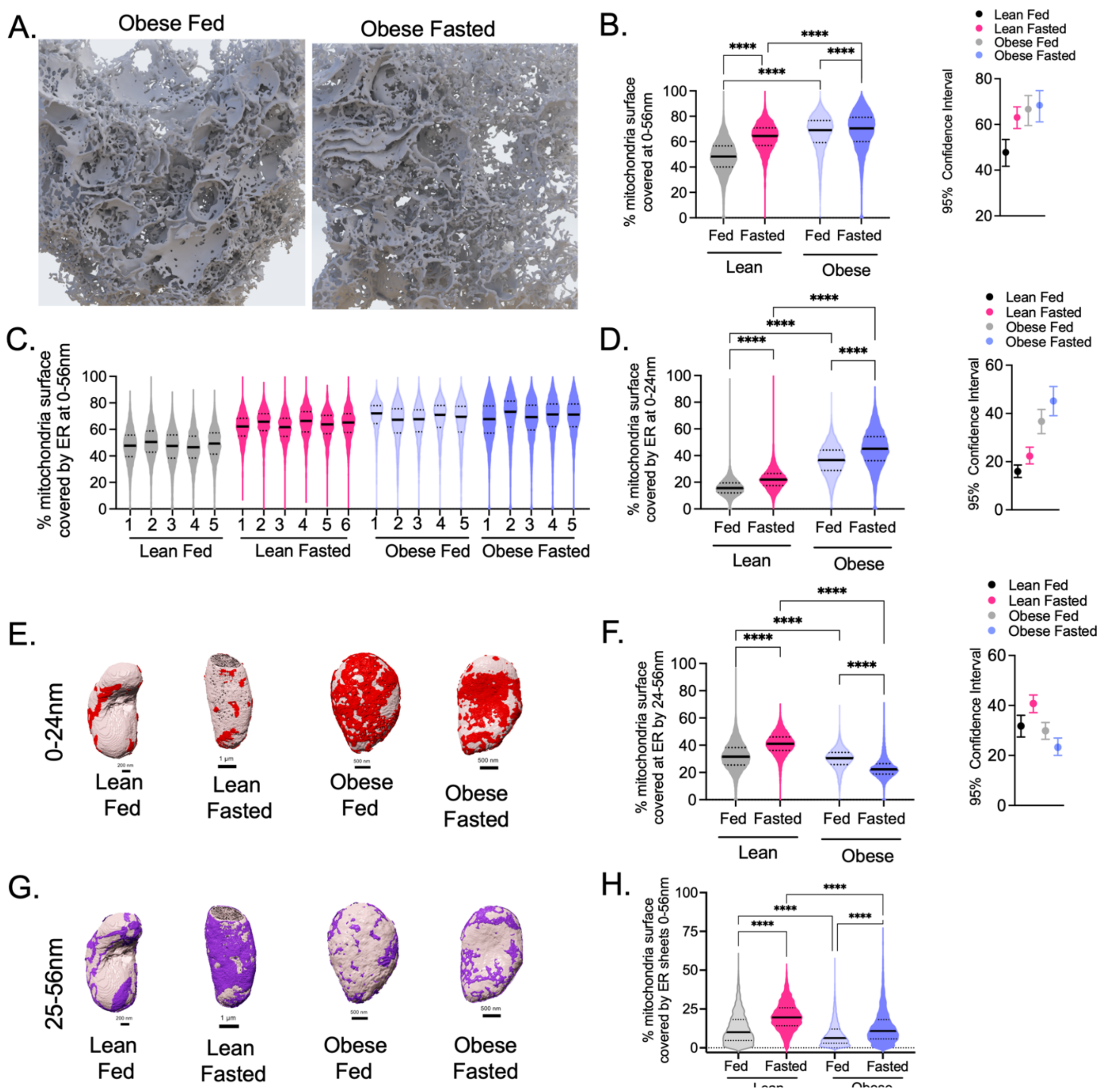

Supplementary Figure 7. Impact of fasting and obesity on hepatic ER and mitochondria interaction. (A) 3D rendering of FIB-SEM data performed in Houdini software showing the ER network in livers from obese mice in Fed (left) and Fasted (right) state. (1,000×1,000×400 voxels, 8×8×3.2 μm^3). (B) Left: Quantification of the percent of mitochondria surface covered by ER at 0-56nm distance in livers from lean and obese mice in fed and fasted state. Right: 95% of confidence interval for data in B. (C) Quantification of the percent of mitochondria surface covered by ER at 0-56nm distance per cell (Lean fed (n = 3000, 3173, 3109, 2787, 2787), lean fasted (n = 894, 1183, 1275, 1246, 757, 1323), obese fed (n= 2084, 5054, 3854, 3521, 3201) and obese fasted (n= 3250, 2781, 2607, 2065, 1999) states. (D) Left: Quantification of the percent of mitochondria surface covered by ER at 0-24nm distance in livers from lean and obese mice in fed and fasted

state. Right: 95% of confidence interval for data in D. (E) Different examples of mitochondria surface covered by ER at 0-24nm distance at indicated conditions. Scale bars from left to right: 200nm, 1µm, 500nm, 500nm. (F) Left: Quantification of the percent of mitochondria surface covered by ER at 24-56nm distance in livers from lean and obese mice in fed and fasted state. Right: 95% of confidence interval for data in F. (G) Different examples of mitochondria surface covered by ER at 24-56nm distance at indicated conditions. Scale bars from left to right: 200nm, 1µm, 500nm, 500nm. (H) Quantification of the percent of mitochondria surface covered by ER sheets at 0-56nm distance in livers from lean and obese mice in fed and fasted state. For B, D, and F, Lean Fed n=14856; Lean Fasted n=6678; Obese Fed n=17714; Obese fasted n=12702. For H, lean fed n=2786, lean fasted n=1275, obese fed n=3854, obese fasted=2607 mitochondria. For B, D, F, H, one-way Anova and Tukey's multiple comparisons test. ****$p<0.0001$.

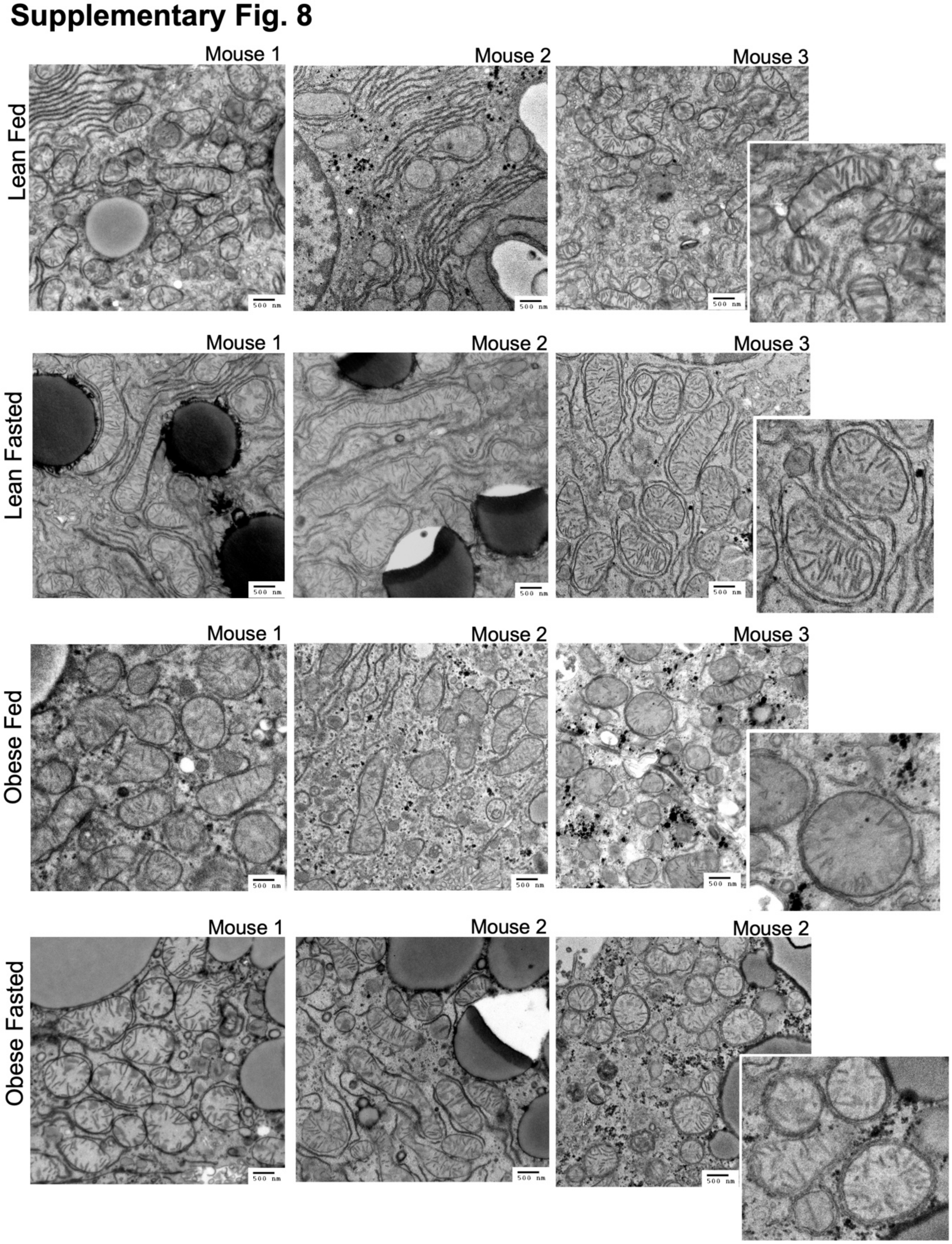

Supplementary Figure 8. TEM analysis of livers from multiple mice under different nutritional or disease states. Representative 2D TEM images of livers from multiple mice under indicated states. Scale bars: 500nm.

Supplementary Fig. 9

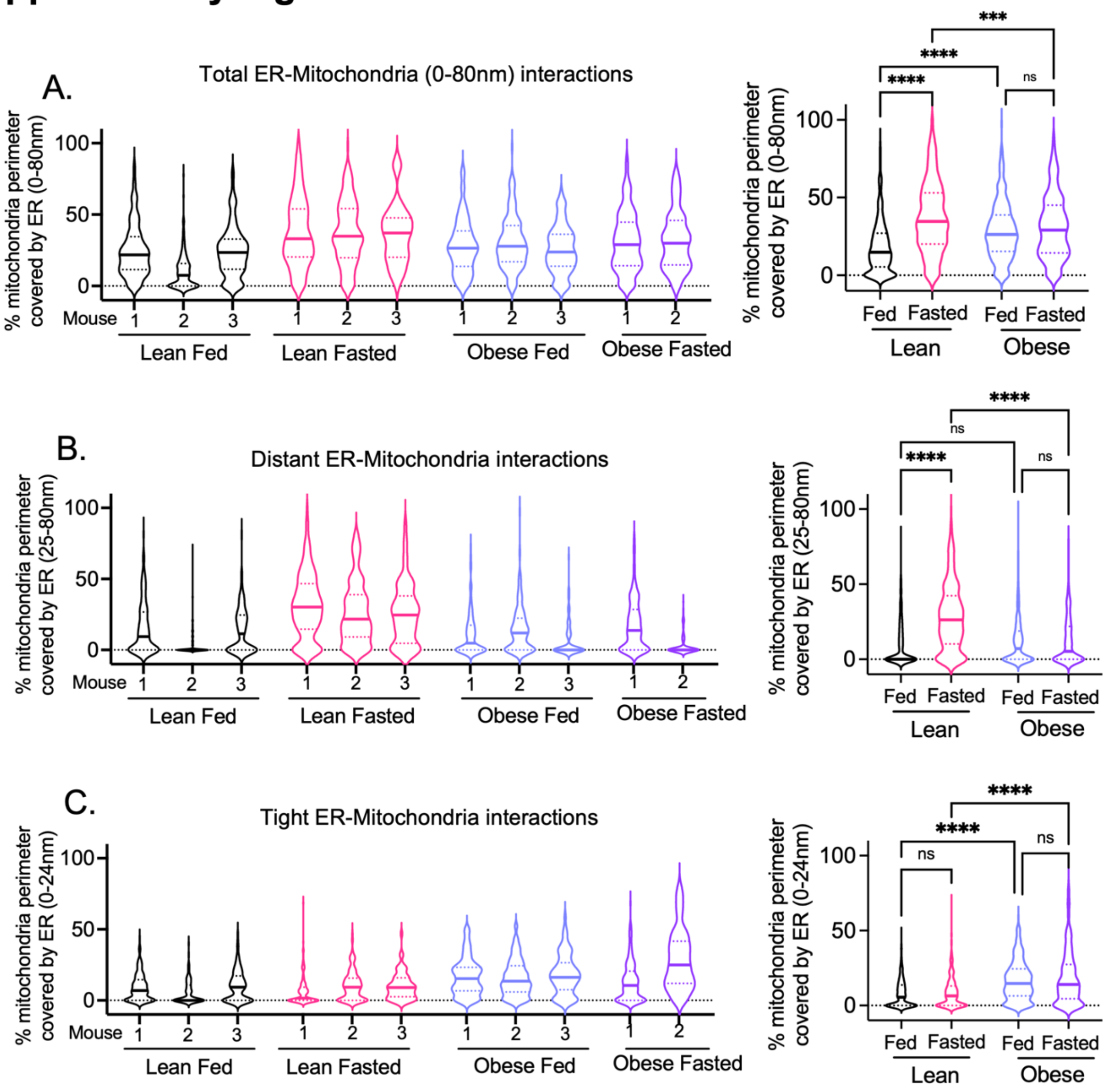

Supplementary Figure 9. Quantification of ER-mitochondria interactions at different distances in 2D TEM images. (A-C) Quantification of ER-mitochondria interactions at 0-80nm (A), 25-80nm (B) and 0-24nm (C) distance, graphed mouse by mouse (left) and grouped per indicated condition (right). Groups depict n=3 lean fed mice, n=3 lean fasted mice, n=3 obese fed mice and n=2 obese fasted mice. For A, B, C, one-way Anova and Tukey's multiple comparisons test. *** p= 0.0001; ****p<0.0001.

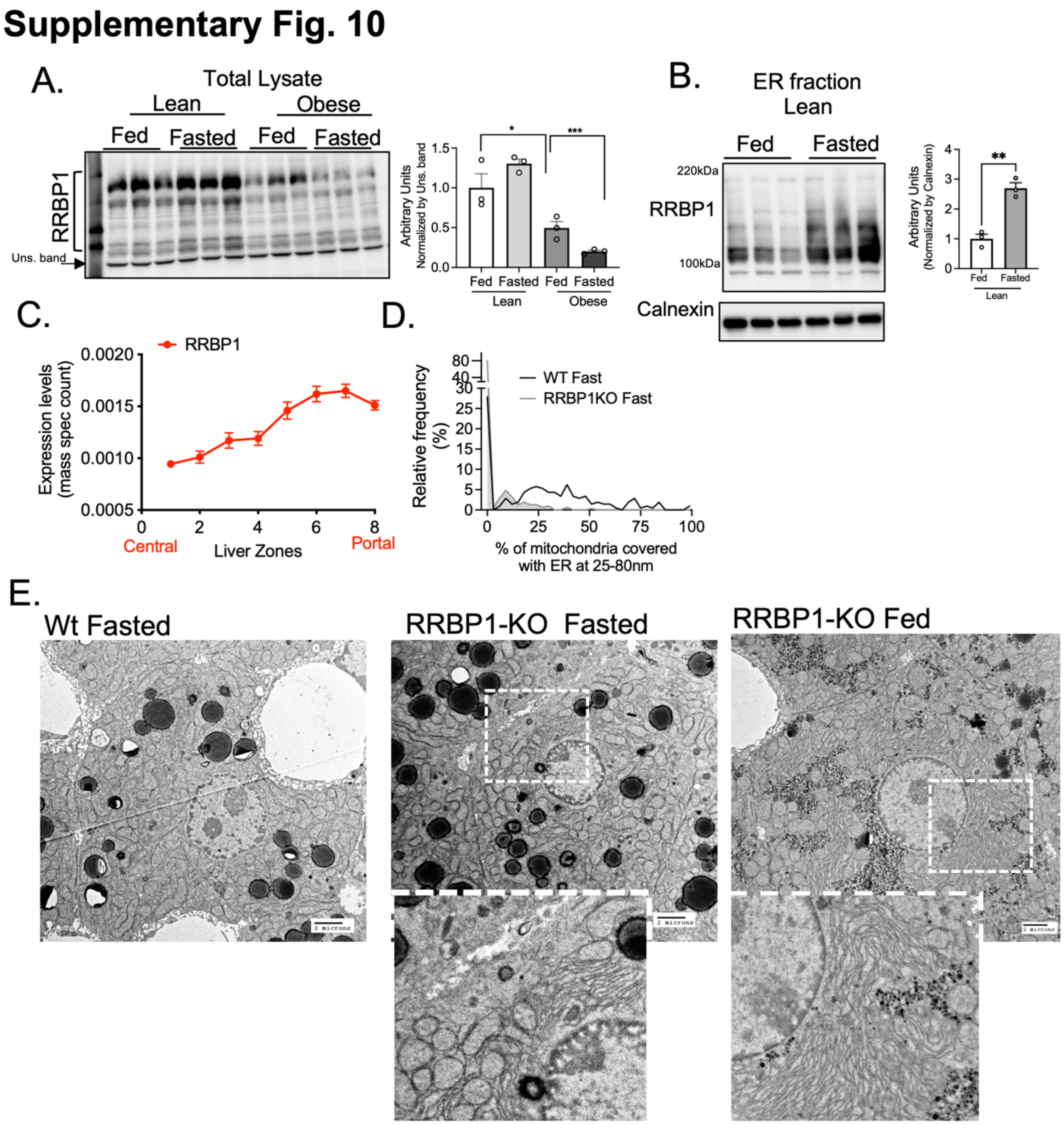

Supplementary Figure 10. Impact of RRBP1 deletion on ER and mitochondria architecture. (A) Immunoblotting and quantification analysis of RRBP1 protein in total liver lysates of indicated conditions. n= 3 in each group. (B) Immunoblotting and quantification analysis of RRBP1 protein in ER fractions from liver of indicated conditions. n= 3 in each group. (C) Mass-spectrometry counts and spatial expression pattern of RRBP1 protein across the liver zone. Data was mined from a published report[42]. (D) Frequency distribution of percent of mitochondria surface covered by ER at 25-80nm distance. (E) Representative TEM images of wild type control and RRBP1 KO mice livers, taken in fasting condition. Scale bars: 2µm. For the bar graphs data are shown as mean ± s.e.m; In A, one-way Anova and Tukey's multiple comparisons test, *p=0.032, **p=0.0024; In B, two-tailed, unpaired t-test. **p<0.002.

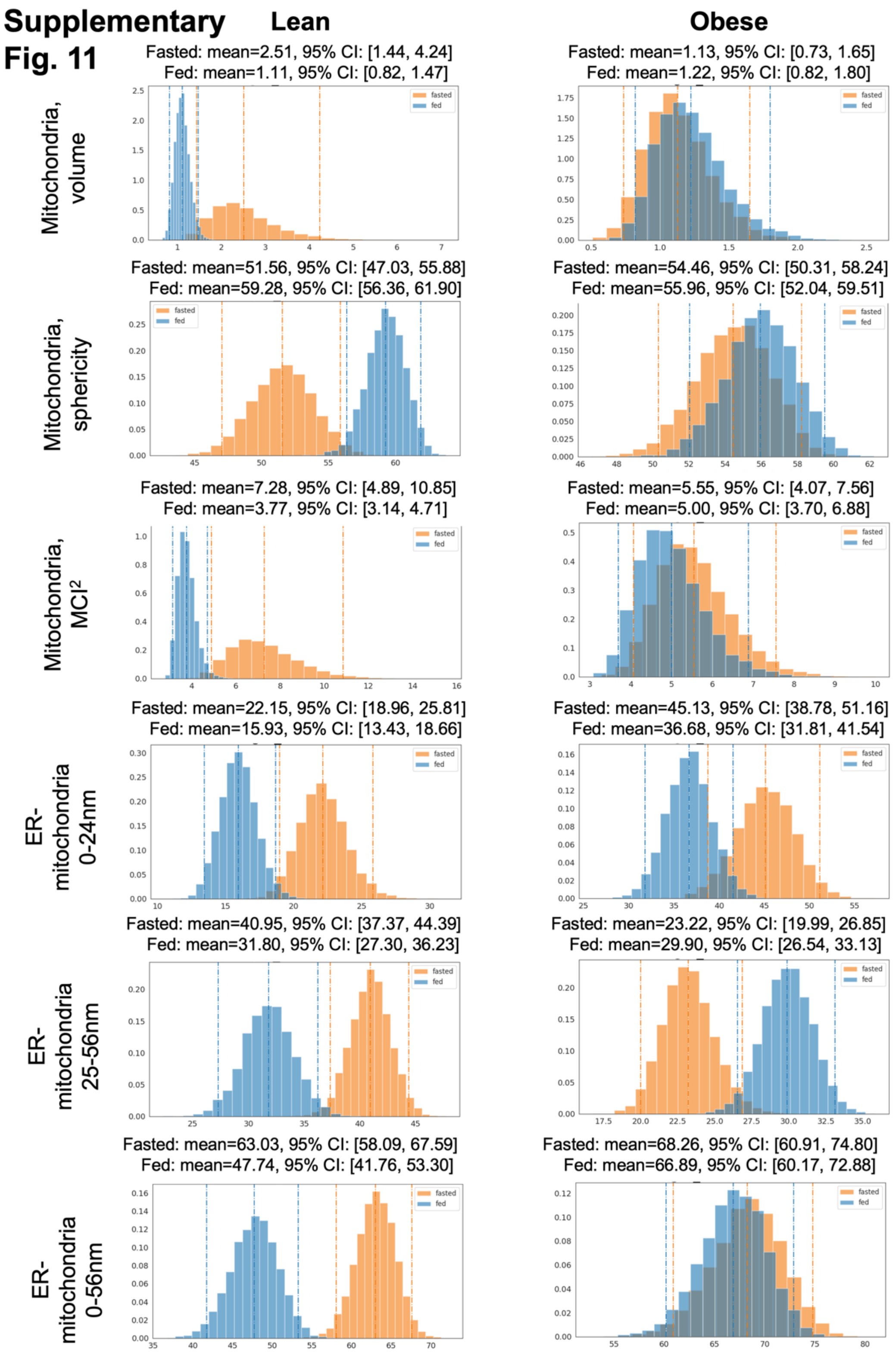

Supplementary Figure 11. Bootstrapped confidence intervals. Bootstrapped confidence intervals of the indicated parameters, showing the distribution and confidence intervals. A detailed explanation of statistical analysis is provided in the Methods section.
