# Supplementary material for: Spatial mapping of hepatic ER and mitochondria architecture reveals zonated remodeling in fasting and obesity
Source: Nat Commun. 2024 May 10;15:3982. doi: 10.1038/s41467-024-48272-7 (PMC11087507; doi:10.1038/s41467-024-48272-7)
Supplement: Supplementary file 3 — Description of Additional Supplementary Files [file 41467_2024_48272_MOESM3_ESM.pdf]

Description of Additional Supplementary Files

Supplementary Movie 1: Raw FIB-SEM volume of the lean fed liver dataset and 3D rendering of all the mitochondria from a single hepatocyte.

Supplementary Movie 2: Raw FIB-SEM volume of the lean fasted liver dataset and 3D rendering of all the mitochondria from a single hepatocyte.

Supplementary Movie 3: Representative video demonstrating mitochondria (pink) – lipid droplet (yellow) contact sites in lean fasted state.

Supplementary Movie 4: Remodeling of ER sheets in fasted state. Rough ER sheets are remodeled as single curved sheets, often surrounding the entire mitochondria volume.

Supplementary Movie 5: Remodeling of hepatocyte subcellular environment in fasted state. Rough ER sheets are remodeled as single curved sheets, often surrounding the entire mitochondria volume.

Supplementary Movie 6: Single mitochondrion (red) and ER (white) at 0-80 nm distance in lean fasted state.

Supplementary Movie 7: Raw FIB-SEM dataset from the periportal region of a lean mouse liver.

Supplementary Movie 8: Raw FIB-SEM dataset from the pericentral region of a lean mouse liver.

Supplementary Movie 9: Single mitochondrion (red) and ER (white) at 0-24 nm distance in obese fed state.
