# Supplementary material for: Spatial mapping of hepatic ER and mitochondria architecture reveals zonated remodeling in fasting and obesity
Source: Nat Commun. 2024 May 10;15:3982. doi: 10.1038/s41467-024-48272-7 (PMC11087507; doi:10.1038/s41467-024-48272-7)
Supplement: Supplementary file 13 — Reporting Summary [file 41467_2024_48272_MOESM13_ESM.pdf]

nature portfolio

Corresponding author(s): Gokhan Hotamisligil, Ana Paula Arruda

Last updated by author(s): Apr 7, 2024

# Reporting Summary

Nature Portfolio wishes to improve the reproducibility of the work that we publish. This form provides structure for consistency and transparency in reporting. For further information on Nature Portfolio policies, see our Editorial Policies and the Editorial Policy Checklist.

## Statistics

For all statistical analyses, confirm that the following items are present in the figure legend, table legend, main text, or Methods section.

| n/a | Confirmed | |
|---|---|---|
| ☐ | ☒ | The exact sample size (*n*) for each experimental group/condition, given as a discrete number and unit of measurement |
| ☐ | ☒ | A statement on whether measurements were taken from distinct samples or whether the same sample was measured repeatedly |
| ☐ | ☒ | The statistical test(s) used AND whether they are one- or two-sided<br>*Only common tests should be described solely by name; describe more complex techniques in the Methods section.* |
| ☒ | ☐ | A description of all covariates tested |
| ☐ | ☒ | A description of any assumptions or corrections, such as tests of normality and adjustment for multiple comparisons |
| ☐ | ☒ | A full description of the statistical parameters including central tendency (e.g. means) or other basic estimates (e.g. regression coefficient) AND variation (e.g. standard deviation) or associated estimates of uncertainty (e.g. confidence intervals) |
| ☐ | ☒ | For null hypothesis testing, the test statistic (e.g. *F*, *t*, *r*) with confidence intervals, effect sizes, degrees of freedom and *P* value noted<br>*Give P values as exact values whenever suitable.* |
| ☒ | ☐ | For Bayesian analysis, information on the choice of priors and Markov chain Monte Carlo settings |
| ☒ | ☐ | For hierarchical and complex designs, identification of the appropriate level for tests and full reporting of outcomes |
| ☒ | ☐ | Estimates of effect sizes (e.g. Cohen's *d*, Pearson's *r*), indicating how they were calculated |

*Our web collection on statistics for biologists contains articles on many of the points above.*

## Software and code

Policy information about availability of computer code

| | |
|---|---|
| Data collection | Nikon NIS Elements v4.51.0 software was used to acquire the confocal data. ZEN 3.0 SR FP2 (Black ed., 64-bit, v16) was used for the acquisition, processing and analysis of Lattice-SIM images. Customized LabVIEW code was used for FIBSEM image acquisition. Scale Invariant Feature Transform (SIFT) based algorithm was utilized for FIBSEM image registration and alignment using IMOD and FiJi Image registration/alignment: IMOD (http://bio3d.colorado.edu/imod/openSource), Fiji (doi: 10.1038/nmeth.2019). |
| Data analysis | We used ariadne.ai 3dEMtrace platform for organelle segmentation, Arivis Vision 4D v3.6 for object creation, reconstructions and data analysis, Houdini (SideFX) v18 for visualization of the data (Videos 5, 6 and 9 only), FiJi for confocal and TEM image analysis, Microsoft Excel v16.83 and Graphpad Prism v10 for data processing and analysis. Please see the materials&methods section for description. The analysis pipeline and code used to analyze the organelle interactions and quantifications in Supplementary Fig. 4F can be found at https://github.com/SabriUlkerCenter |

For manuscripts utilizing custom algorithms or software that are central to the research but not yet described in published literature, software must be made available to editors and reviewers. We strongly encourage code deposition in a community repository (e.g. GitHub). See the Nature Portfolio guidelines for submitting code & software for further information.

## Data

Policy information about availability of data

All manuscripts must include a data availability statement. This statement should provide the following information, where applicable:

- Accession codes, unique identifiers, or web links for publicly available datasets
- A description of any restrictions on data availability
- For clinical datasets or third party data, please ensure that the statement adheres to our policy

The previously published (Parlakgul et al, Nature, 2022) raw FIB-SEM volumes of the lean and obese mice in the fed state can be reached at https://doi.org/10.6019/EMPIAR-10791 . The different features that were extracted and analyzed in this work, such as instance-based mitochondria segmentation and other raw FIB-SEM (Lean fasted, Obese fasted, Obese LacZ, Obese RRBP1, peri-central, peri-portal) and segmentation data deposited to the EMPIAR database and can be reached here: https://doi.org/10.6019/EMPIAR-12017 . Supplementary videos can also be reached at
https://www.youtube.com/playlist?list=PLpzquMkvsJ9WCAl5Uea2W1vzgkHjjxfWL
The uncropped raw versions of the western blots generated during the current study are provided together with the source data. Source data are provided with this paper.

## Research involving human participants, their data, or biological material

Policy information about studies with human participants or human data. See also policy information about sex, gender (identity/presentation), and sexual orientation and race, ethnicity and racism.

| | |
|---|---|
| Reporting on sex and gender | Current study do not involve any human participants, data or their biological material. |
| Reporting on race, ethnicity, or other socially relevant groupings | Current study do not involve any human participants, data or their biological material. |
| Population characteristics | Current study do not involve any human participants, data or their biological material. |
| Recruitment | Current study do not involve any human participants, data or their biological material. |
| Ethics oversight | Current study do not involve any human participants, data or their biological material. |

Note that full information on the approval of the study protocol must also be provided in the manuscript.

# Field-specific reporting

Please select the one below that is the best fit for your research. If you are not sure, read the appropriate sections before making your selection.

☒ Life sciences ☐ Behavioural & social sciences ☐ Ecological, evolutionary & environmental sciences

For a reference copy of the document with all sections, see nature.com/documents/nr-reporting-summary-flat.pdf

# Life sciences study design

All studies must disclose on these points even when the disclosure is negative.

| | |
|---|---|
| Sample size | Sample size was determined based on previous studies (PMID: 35264794, 25419710). For TEM analysis, we used 3 mice per group. For time-course EM analysis, we used 2 mice per time-point. In the FIB-SEM studies, we analyzed all the mitochondria present in the hepatocyte volumes and their interactions with ER. For Lean Fed, Obese Fed, Lean Fasted and Obese Fasted datasets, these analyses were done from 5 separate hepatocyte volumes. For Obese LacZ and Obese RRBP1, it was done for the full dataset volume. Sample size (analyzed mitochondria number) are as follows: n=14,855 (Lean Fed), n=6,678 (Lean Fasted), n=17,721 (Obese Fed) n=12,712 (Obese Fasted), n=23,475 (Obese LacZ), n=29,112 (Obese RRBP1). |
| Data exclusions | No data was excluded. |
| Replication | All replications were successful. Please see figure legends for detailed explanation. |
| Randomization | Mice were randomly allocated to groups. Only criteria were sex and age as explained in the methods. |
| Blinding | Imaging studies could not be done blinded due to the evident intrinsic features of the datasets. In vivo studies could not be blinded due to the adenoviral injection protocol. Experimental and control samples were processed together using the same conditions. |

# Reporting for specific materials, systems and methods

We require information from authors about some types of materials, experimental systems and methods used in many studies. Here, indicate whether each material, system or method listed is relevant to your study. If you are not sure if a list item applies to your research, read the appropriate section before selecting a response.

## Materials & experimental systems

| n/a | Involved in the study |
|---|---|
| ☐ | ☒ Antibodies |
| ☒ | ☐ Eukaryotic cell lines |
| ☒ | ☐ Palaeontology and archaeology |
| ☐ | ☒ Animals and other organisms |
| ☒ | ☐ Clinical data |
| ☒ | ☐ Dual use research of concern |
| ☒ | ☐ Plants |

## Methods

| n/a | Involved in the study |
|---|---|
| ☒ | ☐ ChIP-seq |
| ☒ | ☐ Flow cytometry |
| ☒ | ☐ MRI-based neuroimaging |

# Antibodies

Antibodies used

Western Blots:
RRBP1 - Proteintech (catalog: 22015-1-AP) - 1:1000 dilution
Calnexin - Santa Cruz Biotechnologies (catalog: sc-6465) - 1:1000 dilution
Alpha-tubulin - Proteintech (catalog: 66031-1-Ig)- 1:1000 dilution
Beta-tubulin - Abcam (catalog: ab21058) - 1:1000 dilution
Anti-rabbit IgG, HRP-linked - Cell Signaling (anti-rabbit, catalog: 7074) - 1:5000 dilution
Anti-mouse IgG, HRP-linked - Cell Signaling (anti-mouse, catalog: 7076) - 1:5000 dilution
Anti-goat IgG, HRP-linked - Santa Cruz Biotechnologies (anti-goat, catalog: sc-6465) - 1:5000 dilution

Immunofluorescence:
RRBP1 - Proteintech (catalog: 22015-1-AP) - 1:100 dilution
Glutamine synthase - Santa Cruz Biotechnology (catalog: 74430) - 1:200 dilution
OXPHOS - Abcam (catalog: ab110413) - 1:200 dilution
STED Abberior STAR-Orange and STAR-Red secondary dyes (Abberior catalog: STRED, STORANGE) - 1:1000 dilution
Alexa-fluor secondary antibodies - 1:1000 dilution

Validation

p180 (RRBP1); Proteintech; 22015-1-AP;
Validated in Fig.5A by genetic deletion in mouse liver tissue

Calnexin; Santa Cruz Biotech. sc-6465;
Validated by gain of expression at https://datasheets.scbt.com/sc-6465.pdf

b-tubulin-HRP; Abcam; ab21058;
Validated with 103 citations at https://www.abcam.com/hrp-beta-tubulin-antibody-loading-control-ab21058.html

a-tubulin; Proteintech; 66031-1-Ig;
Validated with 853 citations at https://www.ptglab.com/products/tubulin-Alpha-Antibody-66031-1-Ig.htm

Glutamine synthetase; Santa Cruz Biotechnology; sc-74430;
Validated in PMID: 35771919

OXPHOS; Abcam; ab110413;
Validated with 1024 citations at https://www.abcam.com/products/panels/total-oxphos-rodent-wb-antibody-cocktail-ab110413.html

# Animals and other research organisms

Policy information about studies involving animals; ARRIVE guidelines recommended for reporting animal research, and Sex and Gender in Research

Laboratory animals

All animal experimentation was approved by the Institutional Animal Care and Use Committee at the Harvard T.H. Chan School of Public Health and University of California, Berkeley. The mice were housed at room temperature (70°F) and at 30-70% relative humidity, on a 12h light/dark cycle with free access to water and chow diet (PicoLab Mouse Diet 20 no. 5058, LabDiet) in the Harvard T.H. Chan School of Public Health pathogen-free barrier facility and in University of California, Berkeley pathogen-free barrier facility. The temperature and humidity control was monitored by operation personnel. We used the leptin-deficient B6.Cg-Lepob/J (ob/ob) male mouse (stock no. 000632) as a model of genetic obesity and aged/gender matched C57BL/6J mice (Jackson Labs, stock no. 000664) or ob/+ heterozygotes (Jackson Labs, stock no. 000632) as lean controls. These animals were purchased around 6 weeks of age and used for experimentation between 9–11 weeks of age. RRBP1 heterozygous mice were obtained from the Mutant Mouse Resource and Research Center (MMRRC), NIH (stock no. 051080-JAX). Wild-type and homozygous RRBP1 null mice were obtained by crossing heterozygous mice at the Harvard T.H. Chan School facility and University of California, Berkeley facility.

| | |
|---|---|
| Wild animals | No wild animals were used in the study. |
| Reporting on sex | In the current study, we used male mice. |
| Field-collected samples | No field-collected samples were used in the study. |
| Ethics oversight | All animal experimentation was approved by the Institutional Animal Care and Use Committee at the Harvard T.H. Chan School of Public Health and University of California, Berkeley. |

Note that full information on the approval of the study protocol must also be provided in the manuscript.

## Plants

| | |
|---|---|
| Seed stocks | No plants were used in the current study. |
| Novel plant genotypes | No plants were used in the current study. |
| Authentication | No plants were used in the current study. |
