# Supplementary material for: Spatial mapping of hepatic ER and mitochondria architecture reveals zonated remodeling in fasting and obesity
Source: Nat Commun. 2024 May 10;15:3982. doi: 10.1038/s41467-024-48272-7 (PMC11087507; doi:10.1038/s41467-024-48272-7)
Supplement: Supplementary file 14 — Source Data [file 41467_2024_48272_MOESM14_ESM.zip › Source Data/Uncropped western blots.pptx]

## Slide 1
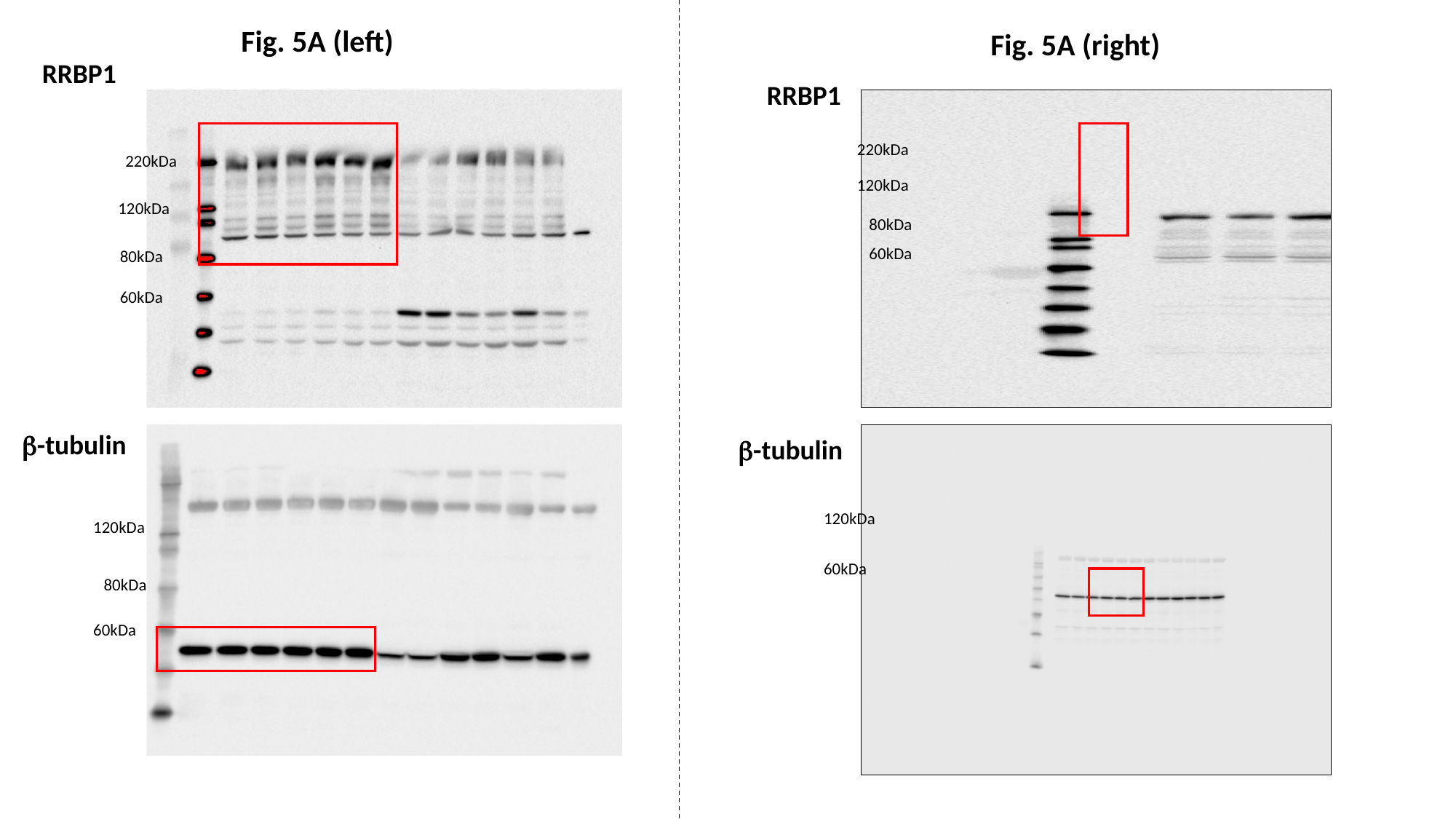

Fig. 5A (left)
Fig. 5A (right)
RRBP1
RRBP1
220kDa
220kDa
120kDa
120kDa
80kDa
60kDa
80kDa
60kDa
b-tubulin
b-tubulin
120kDa
120kDa
60kDa
80kDa
60kDa

## Slide 2
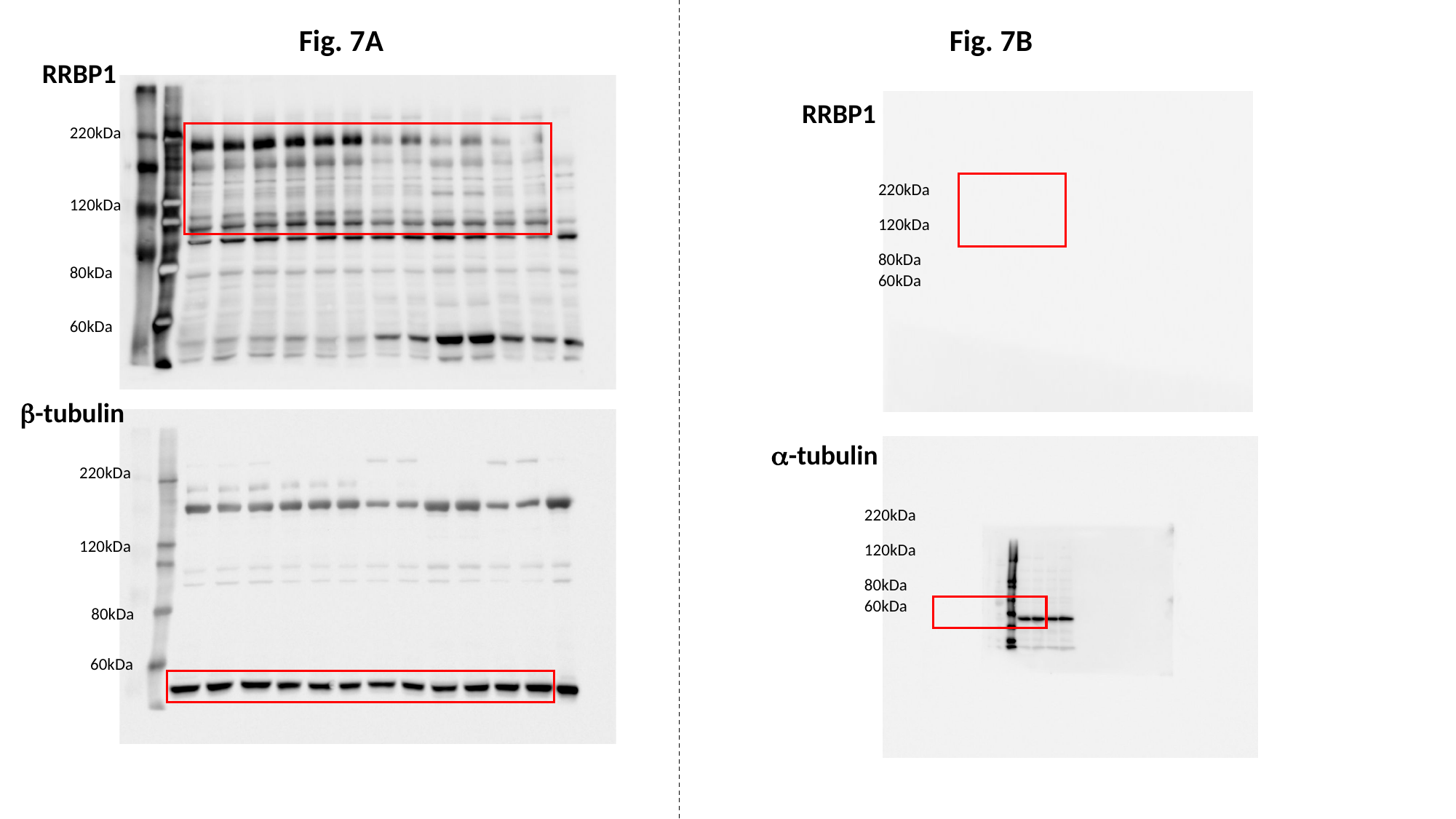

Fig. 7A
Fig. 7B
RRBP1
RRBP1
220kDa
220kDa
120kDa
120kDa
80kDa
80kDa
60kDa
60kDa
b-tubulin
a-tubulin
220kDa
220kDa
120kDa
120kDa
80kDa
60kDa
80kDa
60kDa

## Slide 3
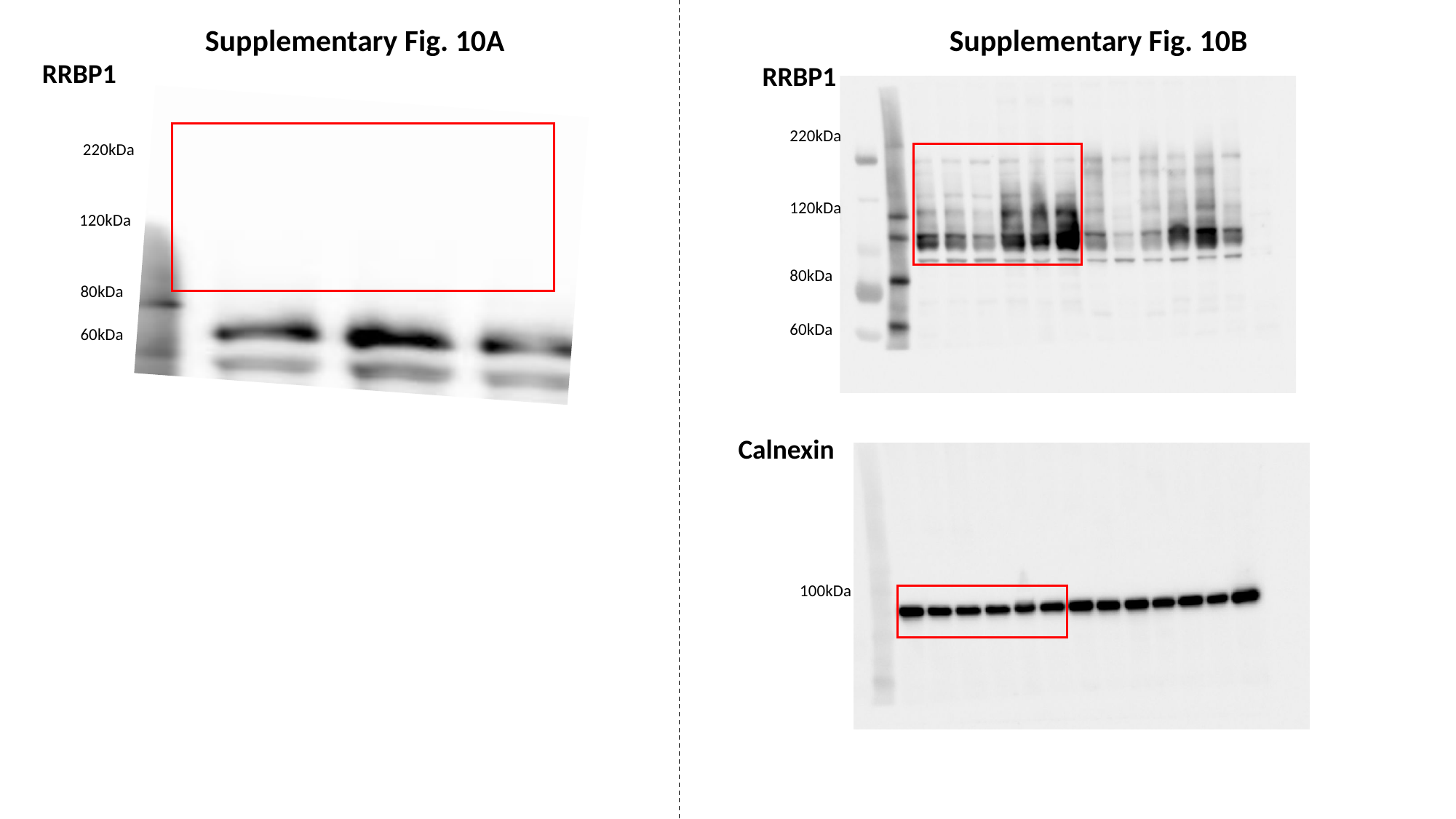

Supplementary Fig. 10A
Supplementary Fig. 10B
RRBP1
RRBP1
220kDa
220kDa
120kDa
120kDa
80kDa
80kDa
60kDa
60kDa
Calnexin
100kDa
